# Microbiological contamination of mobile phones of clinicians in intensive care units and neonatal care units in public hospitals in Kuwait

**DOI:** 10.1186/s12879-015-1172-9

**Published:** 2015-10-15

**Authors:** Mohammed Heyba, Mohammad Ismaiel, Abdulrahman Alotaibi, Mohamed Mahmoud, Hussain Baqer, Ali Safar, Noura Al-Sweih, Abdullah Al-Taiar

**Affiliations:** Department of Community Medicine and Behavioral Sciences, Faculty of Medicine, Kuwait University, Box: 24923, Safat, 13110 Kuwait; Department of Microbiology, Faculty of Medicine, Kuwait University, Box: 24923, Safat, 13110 Kuwait

**Keywords:** Mobile phones, Hygiene, Contamination, Colonization, Middle East, Kuwait

## Abstract

**Background:**

The objective of this study was to explore the prevalence of microbiological contamination of mobile phones that belong to clinicians in intensive care units (ICUs), pediatric intensive care units (PICUs), and neonatal care units (NCUs) in all public secondary care hospitals in Kuwait. The study also aimed to describe mobile phones disinfection practices as well as factors associated with mobile phone contamination.

**Methods:**

This is a cross-sectional study that included all clinicians with mobile phones in ICUs, PICUs, and NCUs in all secondary care hospitals in Kuwait. Samples for culture were collected from mobile phones and transported for microbiological identification using standard laboratory methods. Self-administered questionnaire was used to gather data on mobile phones disinfection practices.

**Results:**

Out of 213 mobile phones, 157 (73.7 %, 95 % CI [67.2–79.5 %]) were colonized. Coagulase-negative staphylococci followed by *Micrococcus* were predominantly isolated from the mobile phones; 62.9 % and 28.6 % of all mobile phones, respectively. Methicillin-resistant *Staphylococcus aureus* (MRSA) and Gram-negative bacteria were identified in 1.4 % and 7.0 % of the mobile phones, respectively. Sixty-eight clinicians (33.5 %) reported that they disinfected their mobile phones, with the majority disinfecting their mobile phones only when they get dirty. The only factor that was significantly associated with mobile phone contamination was whether a clinician has ever disinfected his/her mobile phone; adjusted odds ratio 2.42 (95 % CI [1.08–5.41], *p*-value = 0.031).

**Conclusion:**

The prevalence of mobile phone contamination is high in ICUs, PICUs, and NCUs in public secondary care hospitals in Kuwait. Although some of the isolated organisms can be considered non-pathogenic, various reports described their potential harm particularly among patients in ICU and NCU settings. Isolation of MRSA and Gram-negative bacteria from mobile phones of clinicians treating patients in high-risk healthcare settings is of a major concern, and calls for efforts to consider guidelines for mobile phone disinfection.

## Background

Healthcare-associated infections (HAIs) are a major challenge to the healthcare system and are associated with significant mortality, morbidity, and high economic burden. It is estimated that of every 100 hospitalized patients at any given time, seven in developed and ten in developing countries will acquire at least one HAI [1]. HAIs are becoming increasingly common due to the expansion of the population at risk, which results from aging population, increase of chemotherapeutic options for cancer treatment, increase in the number of patients with transplants, in addition to complex and invasive surgical and medical care procedures that are increasingly being provided in acute and non-acute-care settings [2]. Patients in intensive care units (ICUs) are particularly susceptible to HAIs because of their poor health status in addition to the use of invasive equipment like catheters and cannulae. Similarly, infants in neonatal care units (NCUs) have a higher risk of HAIs because of their immature immune systems, their skin does not provide a strong barrier against organisms in the environment and a large number of these infants are premature and often require invasive procedures to sustain their life such as mechanical ventilation and total parenteral nutrition [3].

Contaminated hands of healthcare providers play a major role in spreading infections in healthcare settings. Hand hygiene is one of the most important preventive interventions against the spread of infections in healthcare settings [4]. Objects with frequent hand contact can serve as reservoirs from which infections can spread to the hands of healthcare providers and then to patients. Examples of these objects include medical equipment like stethoscopes and other accessories such as mobile phones [5, 6]. Mobile phones have become an indispensable accessory of today’s society, and they are being used extensively in hospital settings. They are commonly handled irrespective of the cleanliness of hands and rarely disinfected, thus may harbor pathogenic bacteria [7].

The debate on the restriction of mobile phone use in clinical settings due to electromagnetic interference that may affect medical equipment has reached an end; but the potential role of mobile phones in transmitting infection remains under intense debate. Several studies have described the contamination of clinicians’ mobile phones in healthcare settings, and reported a level of contamination and type of bacteria that depend on the clinical and geographical setting [8]. Studies that investigated the contamination of clinicians’ mobile phones in developed countries, like USA and UK, reported a level of overall mobile phone contamination (pathogenic and non-pathogenic organisms) ranging from 75 % to 96 % [5, 8–10]. The most common isolated organisms were coagulase-negative staphylococci (CoNS) and *Micrococcus*; while between 9 % and 25 % of mobile phones were contaminated by other pathogenic bacteria known to cause HAIs, including methicillin-sensitive and methicillin-resistant *Staphylococcus aureus* (MSSA & MRSA), *Acinetobacter* species, and *Pseudomonas* species [5, 8–10]. In addition, studies in healthcare settings in developing countries, including India, Nigeria, and Turkey, demonstrated that 42 % to 97 % of clinicians’ mobile phones are contaminated. CoNS were the most common isolated organisms; while other microorganisms, such as *Escherichia coli*, *Acinetobacter* species, *Pseudomonas* species, and MRSA, were isolated from 8 % to 31 % of the clinicians’ mobile phones [8, 11–15].

Few studies have investigated the contamination of clinicians’ mobile phones in the Middle East. In Saudi Arabia, three studies in healthcare settings, including wards and ICUs, have shown that 43.6 % to 96.5 % of mobile phones that belong to clinicians were contaminated by bacteria or other microorganisms. The most common isolated organisms were also CoNS but 8 % to 14 % of the clinicians’ mobile phones harbored other organisms known to cause HAIs, including *Staphylococcus aureus*, *Enterococcus*, and Gram-negative bacilli [16–18]. In Kuwait, only one small study in one hospital has attempted to describe the contamination of clinicians' mobile phones. This study examined the bacterial profile of 82 mobile phones belong to 82 conveniently selected clinicians in various wards and did not focus on settings with vulnerable patients for infections, such as patients in ICUs and NCUs [19]. In this study we aimed to investigate the prevalence of contamination of mobile phones of clinicians in ICUs, PICUs, and NCUs, describe the microbiological profile of contaminated mobile phones in ICUs, PICUs, and NCUs and investigate the factors associated with mobile phone contamination.

## Methods

### Study hospitals, study design and study participants

This is a cross-sectional study that was conducted in all intensive care units (ICUs) and pediatric intensive care units (PICUs) in all public secondary care hospitals in Kuwait, in addition to all public neonatal care units (NCUs) in the country. There are eleven ICUs distributed in six public secondary care hospitals of which five are PICUs, in addition to four public NCUs located in four hospitals. There are approximately 261 clinicians working in ICUs, PICUs, and NCUs, providing services in three shifts. Facilities for hand hygiene are widely available, including hand sanitizer and alcohol rub beside every bed and incubator. There is no specific policy that bans using mobile phones in ICUs, PICUs or NCUs, although some posters that ask clinicians, patients, and visitors to refrain from carrying their mobile phones into the ward were observed in some units.

**Fig. 1:**
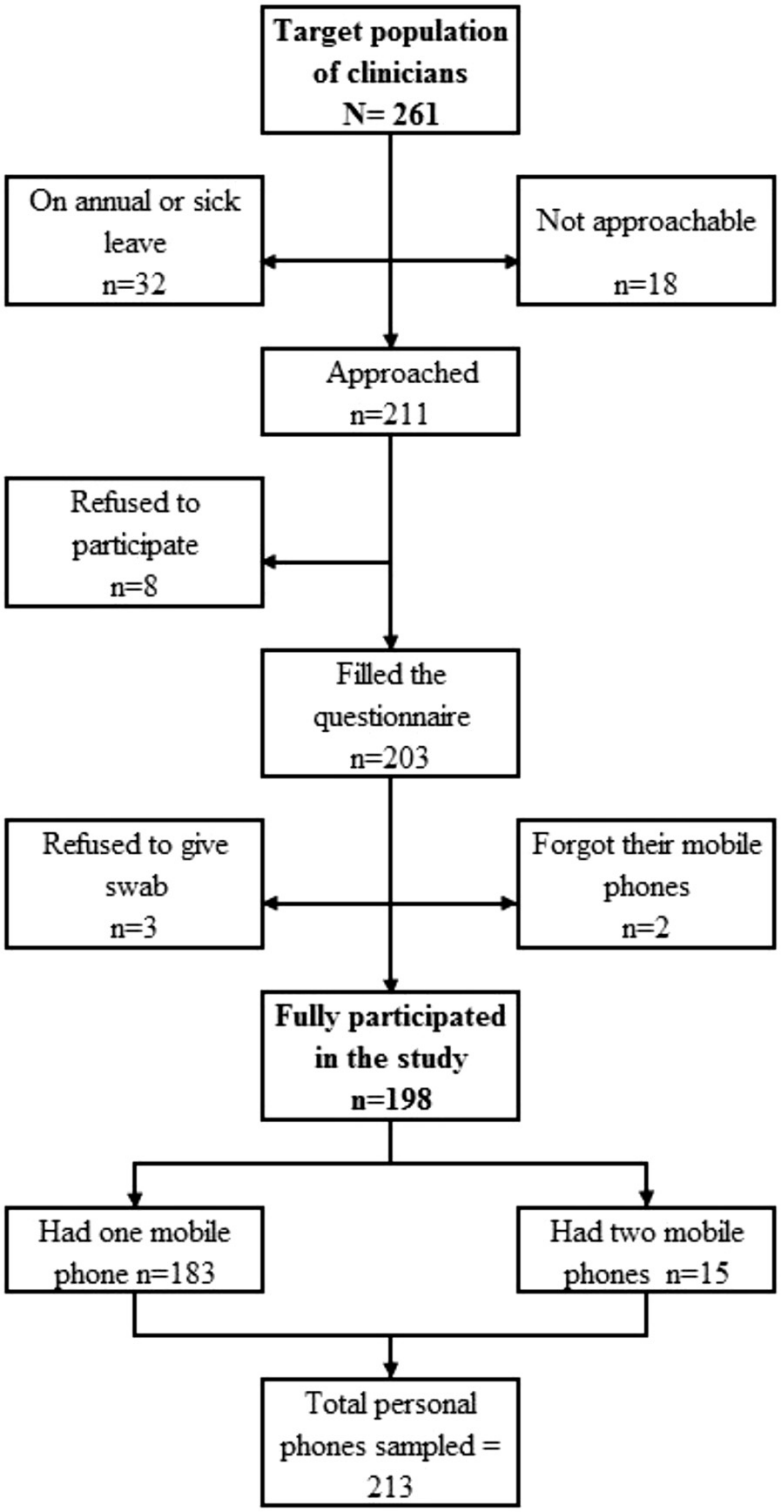
Flow diagram for the clinicians who participated in the study in ICUs, PICUs, and NCUs in public secondary care hospitals in Kuwait, 2013

All clinicians (trainees, assistant registrars, registrars, senior registrars, specialists, senior specialists, and consultants) in ICUs, PICUs, and NCUs were targeted. The only exclusion criterion was the lack of mobile phone ownership. A list of clinicians in each ICU, PICU or NCU was sought, and attempts were made to approach clinicians during their working shift. A second, third, and fourth attempt was made to approach those clinicians that were not available during their working shift. Of all 261 clinicians working in these units, 32 were on annual or sick leave, and another 18 were not approachable despite the four attempts that were made to reach them. In total, 211 clinicians were approached and invited to participate, and 203 of them agreed to participate in the study (response rate = 96.2 %), see Fig. 1. A written informed consent was taken from each participating clinician. The study was approved by the Standing Committee for Coordination of Health and Medical Research in the Ministry of Health, Kuwait.

### Data collection and laboratory methods

A self-administered questionnaire was used to collect data on the participant’s demographics, including age, gender, and nationality in addition to questions regarding their use of mobile phones at work and their perception of the potential role of clinicians’ mobile phones in spreading infections in hospital settings. The questionnaire also included questions on mobile phone hygiene practices; including the frequency of mobile phone disinfection and the disinfectant which clinicians used to clean their mobiles. The questionnaire was pre-tested on twelve clinicians working outside ICUs, PICUs, and NCUs.

While the clinician was filling the questionnaire, swabs were taken from his/her mobile phone(s) for culture. Clinicians were asked whether they carry more than one mobile phone, and if so, separate swabs were taken from each mobile phone (15 participating clinicians had two mobile phones). Swabs were also taken from all shared mobile phones assigned for general use by clinicians who were on-call. In total, 217 mobile phones were sampled (213 personal mobile phones and 4 shared mobile phones), see Fig. 1.

Six senior medical students were trained by a senior microbiologist to obtain the swabs in a standardized manner. The screen, sides, and back of mobile phones were swabbed using a sterile swab. In case of mobile phones with covers, the swab was taken from the outer surfaces of the cover in addition to the screen of the mobile phone. In order to prevent cross contamination, alcohol sanitizer was used to disinfect the hands of the data collectors before swabbing each mobile phone. The collected samples were given unique identification numbers and were labeled with the hospital name and the name of the unit. The samples were kept in transport media and transported to the laboratory for culture within 24 hours of sampling. Swabs were then sub-cultured on blood and chocolate agar plates, which were incubated for 48 hours at 37 °C in 5 % CO_2_. Plates which showed no growth were reported as negative, while those showing any growth were reported as positive. Positive growths were subsequently identified using routine microbiological methods. Sensitivity to antibiotics, including penicillins, cephalosporins, aminoglycosides, erythromycin, meropenem, ciprofloxacin, tetracycline, and chloramphenicol, was tested for Gram-negative organisms [20]. Sensitivity to methicillin and vancomycin was also tested for *Staphylococcus aureus.* The laboratory data included the names of the organisms identified and the pattern of growth, which was indicated by the number of colony forming units on the culture for each organism.

### Statistical analysis

Data were entered and analyzed using Statistical Package for Social Sciences (SPSS). We have calculated 95 % confidence intervals (95 % CI) using exact binomial distribution and conducted various statistical tests despite the fact that we tried to enroll the whole study population. While this may be deemed as inappropriate given the finite nature of the target population (clinicians working in ICUs, PICUs, and NCUs), the fact that trainees, registrars, and other staff rotate between ICUs and other wards in hospitals makes this population dynamic and under constant change. Thus, despite the attempt to recruit the whole study population, the study participants can be considered as a sample of clinicians working in ICUs, PICUs, and NCUs. We used Chi-squared test or Fisher's exact test, to investigate differences in categorical variables. We used univariate and multivariate logistic regression to investigate factors associated with contamination of mobile phones. Likelihood ratio test was used to investigate the association between each factor and mobile phones contamination, comparing model with and without the variable.

## Results

The socio-demographic characteristics, professional rank, and workload of the study participants are shown in Table 1. The mean (SD) of age was 39 (8.7) years. Of 203 clinicians, 155 (76.4 %) were males, and non-Kuwaiti Arabs were the majority of the study participants, 131 (64.5 %). More than half of the clinicians (59.1 %) were registrars, and 126 (62.0 %) examine on average less than ten patients per day. Table 2 shows the pattern of use of mobile phones among the study participants. Of 203 clinicians, 15 (7.4 %) use more than one mobile phone. Approximately one fifth reported that they always answer their mobile phone calls while in the ICU, PICU or NCU. More than half of the participants reported using their mobile phones to search for medical information and/or take photos of the cases. Only 68 (33.5 %) clinicians reported that they have ever disinfected their mobile phones. Of those clinicians, about half (32 clinicians) reported disinfecting their mobile phones daily or weekly, while 28 (41.1 %) clinicians disinfect their mobile phones only when they get dirty. Thirty-two (47.1 %) clinicians reported disinfecting their mobile phones one week before the time of the study. When the 68 clinicians, who reported cleaning or disinfecting their mobile phones, were asked about the way used in mobile phone disinfection, 50 (73.5 %) reported using alcohol wipes and only 9 (13.2 %) used liquid personal hand disinfectant.

**Table 1:** ^a^Socio-demographic characteristics, professional rank and workload of 203 clinicians in ICUs, PICUs, and NCUs in public secondary care hospitals in Kuwait, 2013

Characteristics	
Age^b^ (years)	Mean (standard deviation)
	39.1 (8.7)
Gender	*n* (%)
Male	155 (76.4)
Nationality	*n* (%)
Kuwaiti	30 (14.8)
Non-Kuwaiti: Arab	131 (64.5)
Non-Kuwaiti: Non-Arab	42 (20.7)
Professional rank	*n* (%)
Consultant/senior specialist/specialist/senior registrar	67 (33.0)
Registrar	120 (59.1)
Assistant registrar/trainee	16 (7.9)
Number of patients examined per day	*n* (%)
Less than 10	126 (62.0)
10 to 15	42 (20.7)
More than 15	35 (17.3)

^a^ICUs: intensive care units; PICUs: pediatric intensive care units; NCUs: neonatal care units

^b^Missing for 18 participants

**Table 2:** ^a^Pattern of mobile phone use among 203 clinicians in ICUs, PICUs, and NCUs in public secondary care hospitals in Kuwait, 2013

Use more than one mobile phone (yes)		
		*n* (%)
		15 (7.4)
Answering mobile phone calls:		
	In the ICU, PICU or NCU	*n* (%)
	Never	15 (7.4)
	Sometimes	145 (71.4)
	Always	43 (21.2)
	In other areas in the hospital	*n* (%)
	Never	2 (1.0)
	Sometimes	99 (48.8)
	Always	102 (50.2)
Use of mobile phone at bedside to:		*n* (%)
	Search for medical information	113 (55.7)
	Take photos of cases	111 (54.7)

^a^ICUs: intensive care units; PICUs: pediatric intensive care units; NCUs: neonatal care units

There were 15 clinicians who had two mobile phones at the time of the enrollment. Separate swabs were taken from each mobile phone. There were also two clinicians who forgot their mobile phones at home, and another three clinicians refused taking swabs from their mobile phones despite filling the questionnaire (Fig. 1). Thus, in total we have taken swabs from 213 mobile phones that belong to 198 clinicians. Out of these 213 mobile phones, 157 (73.7 %; 95 % CI [67.2–79.5 %]) were contaminated. Excluding physicians who carried two mobile phones at the time of the study from this analysis, did not change the results materially (73.2 %; 95 % CI [66.5–79.3 %]). Table 3 presents the microbiological profile of 213 mobile phones of 198 clinicians in ICUs, PICUs, and NCUs in public hospitals. CoNS were the most common organisms isolated from the mobile phones, with 134 (62.9 %) mobile phones being contaminated. Other frequently isolated bacteria included *Micrococcus*, with 61 (28.6 %) mobile phones being contaminated. MRSA was identified in three (1.4 %) mobile phones while *Acinetobacter* species were isolated from six (2.8 %) mobile phones. We have also taken swabs from four mobile phones provided by the units and shared by clinicians who were on-call. Three of these four mobile phones were contaminated (three by CoNS, one by *Micrococcus*, and one by *Moraxella osloensis)* (data not shown).

**Table 3:** ^a^Microbiological profile of 213 mobile phones that belong to 198 clinicians in ICUs, PICUs, and NCUs in public secondary care hospitals in Kuwait, 2013

		Positive	No. of colonies
		*n* (%)	Median (IQR)
Overall		157 (73.7)	
CoNS^b^		134 (62.9)	4 (2–10)
*Streptococcus viridans*		15 (7.0)	2 (1–6)
*Staphylococcus aureus*	MSSA^c^	1 (0.5)	-
	MRSA^d^	3 (1.4)	3 (−)
*Micrococcus*		61 (28.6)	3 (2–12)
Diphtheroids		6 (2.8)	60.5 (11–116)
*Bacillus* species		3 (1.4)	-
Other Gram-positive bacilli^e^		15 (7.0)	2 (1–3)
*Escherichia coli*		1 (0.5)	-
*Pantoea* species		2 (0.9)	3.5 (−)
*Acinetobacter* species^f^		6 (2.8)	2 (1–22)
*Moraxella osloensis*		2 (0.9)	2.5 (−)
*Pseudomonas stutzeri*		2 (0.9)	4.5 (−)
*Sphingomonas paucimobilis*		2 (0.9)	-
Fungus		2 (0.9)	-

^a^ICUs: intensive care units; PICUs: pediatric intensive care units; NCUs: neonatal care units; IQR: interquartile range

^b^CoNS: coagulase-negative staphylococci

^c^MSSA: methicillin-sensitive *Staphylococcus aureus*

^d^MRSA: methicillin-resistant *Staphylococcus aureus*

^e^Other than diphtheroids and *Bacillus* species

^f^ *Acinetobacter lwoffii* (2 mobile phones), *Acinetobacter baumannii* (3 mobile phones), both (1 mobile phone)

We used logistic regression to investigate the relationship between mobile phone contamination and various factors. The outcome in the regression model was whether the mobile phone was contaminated or not, regardless of the colonizing organism or the pattern of growth. Mobile phones contaminated by any organism at any growth rate were regarded as positive, while mobile phones that showed no growth of any organism were regarded as negative. Clinicians with two mobile phones were considered to have a non-contaminated mobile phone if neither mobile phone showed microbial growth. Table 4 shows the association between mobile phones contamination and several factors, including the socio-demographic factors, workload, and mobile phone hygiene practices among the 198 clinicians in univariate analysis. Mobile phones contamination was found non-significantly higher in NCUs (79.6 %) compared to ICUs and PICUs (72.1 % and 65.9 % respectively, *p*-value = 0.213). There was no significant difference in the contamination of mobile phones between male and female clinicians (76.2 % vs. 68.1 %, respectively *p*-value = 0.276). Although not statistically significant, mobile phone contamination remained consistently higher in NCUs compared to ICUs and PICUs after stratification by gender, hospital, and the average number of patients seen by clinicians. The only factor that showed significant association with mobile phone contamination in univariate analysis was whether the clinicians have ever disinfected their mobile phones; crude odds ratio 2.054 (95 % CI [1.064−3.963]; *p*-value = 0.033). This was the only factor significantly associated with mobile phone contamination in multivariate analysis, adjusted odds ratio 2.42 (95 % CI [1.08−5.41]; *p*-value = 0.031).

**Table 4:** ^a^Association between microbiological contamination of clinicians’ mobile phones and socio-demographic factors, workload, and mobile phone hygiene practices among 198 clinicians in ICUs, PICUs, and NCUs in public secondary care hospitals in Kuwait, 2013

		Total	Contamination			
		n	Prevalence %	Unadjusted Odds Ratio	95% CI	*p*
Hospital^b^						0.126
	Al-Adan	49	61.2	0.36	[0.12−1.11]	
	Al-Farwaniya	43	81.4	0.99	[0.29−3.43]	
	Al-Jahra	25	84.0	1.19	[0.28−5.06]	
	Al-Sabah	22	63.6	0.40	[0.11−1.46]	
	Mubarak Al-Kabeer	24	70.8	0.55	[0.15−2.05]	
	Maternity	27	81.5	1	Reference	
Unit						0.213
	ICU	61	72.1	0.66	[0.31−1.41]	
	PICU	44	65.9	0.50	[0.22−1.11]	
	NCU	93	79.6	1	Reference	
Gender						0.276
	Male	151	76.2	1.50	[.73−3.07]	
	Female	47	68.1	1	Reference	
Age		181		1.00	[0.96−1.04]	0.982
Nationality						0.167
	Kuwaiti	30	80.0	2.40	[0.80−7.21]	
	Non-Kuwaiti: Arab	128	76.6	1.96	[0.92−4.19]	
	Non-Kuwaiti: non-Arab	40	62.5	1	Reference	
Rank						0.619
	Consultant/senior specialist/specialist/senior registrar	66	77.3	0.85	[0.212−3.413]	
	Registrar	117	71.8	0.64	[0.17−2.40]	
	Assistant registrar/trainee	15	80.0	1	Reference	
Number of patients examined per day						0.569
	Less than 10 patients	124	71.8	0.68	[0.21−2.19]	
	10 to 15 patients	40	75.0	0.80	[0.22−2.98]	
	15 to 20 patients	15	86.7	1.73	[0.27−11.05]	
	More than 20 patients	19	78.9	1	Reference	
Answering mobile phone calls inside ICU, PICU or NCU						0.387
	Never	12	58.3	0.56	[0.15−2.11]	
	Sometimes	143	76.2	1.28	[0.59−2.78]	
	Always	42	71.4	1	Reference	
Ever disinfected mobile phone						0.033
	No	133	78.9	2.05	[1.06−3.96]	
	Yes	65	64.6	1	Reference	
Believe mobile phones play role in spread of infection						0.607
	No	71	71.8	0.84	[0.44−1.62]	
	Yes	125	75.2	1	Reference	
Support banning mobile phones use in ICUs, PICUs or NCUs						0.291
	No	138	71.7	0.68	[0.32−1.4]	
	Yes	57	78.9	1	Reference	

^a^ICUs: intensive care units; PICUs: pediatric intensive care units; NCUs: neonatal care units

^b^We swabbed 8 mobile phones from 8 clinicians in Al-Amiri hospital; all of them were positive for contamination

## Discussion

This study aimed to investigate the microbiological contamination of mobile phones of clinicians in ICUs, PICUs, and NCUs in public secondary care hospitals in Kuwait. Approximately, 74 % of mobile phones that belong to clinicians in ICUs, PICUs, and NCUs were contaminated. This is higher than that reported from Saudi Arabia, where 43.6 % of clinicians’ mobile phones in wards, emergency rooms, out-patient departments, and operating rooms were contaminated [18]; and in India, where 42 % of clinicians’ mobile phones in different wards were contaminated [13]. On the other hand, the prevalence of contamination of clinicians’ mobile phones in our setting was lower than that reported from other studies in Turkey, where 94.5 % of clinicians’ mobile phones in operating rooms and ICUs were contaminated [11], and 97.8 % of clinicians’ mobile phones in all departments were contaminated [12]. Higher estimates of the contamination of clinicians’ mobile phones have also been reported from UK (96.2 % of mobile phones of all physicians) [9], Austria (95 % of mobile phones of anesthetists) [21], Saudi Arabia (96.5 % of mobile phones of clinicians in ICU) [17] and Nigeria (94.6 % of mobile phones of health care workers in a hospital) [15]. While the direct comparison between the findings of different studies is hindered by various factors, including targeting different hospital wards and different laboratory procedures, the contamination rate of clinicians’ mobile phones in Kuwait seems to be within the range that was reported in other literature.

Although not statistically significant, the contamination rate was higher in NCUs (79.6 %) compared to those in ICUs (72.1 %) and PICUs (65.9 %). This remains evident even after adjusting for other factors using logistic regression. It is not clear why clinicians’ mobile phones in NCUs tended to have higher contamination rate than ICUs and PICUs, but it is worth noting that the rate of late-neonatal infections in Kuwait is extremely high, and that it resembles the one in low-income countries [22].

A major objective of our study was to describe the microbiological profile of contaminated mobile phones of clinicians working in ICUs, PICUs, and NCUs. Most mobile phones were colonized with non-pathogenic bacteria, especially those bacteria that constitute the normal flora of the skin, such as CoNS, *Micrococcus*, and diphtheroids. This is similar to other studies which reported CoNS followed by *Micrococcus* as the most common organisms isolated from clinicians’ mobile phones in clinical settings [8]. CoNS have relatively low virulence, but are becoming increasingly recognized as the most common cause of nosocomial bacteraemia associated with indwelling devices [23]. Despite the fact that CoNS are considered non-pathogenic in normal circumstances, their presence in high levels on objects with frequent hand contact like mobile phones in settings like ICUs may pose a risk of bacteraemia in immunocompromised patients[9]. In addition, CoNS are identified as the most common cause of late-onset neonatal sepsis in developed countries [24–26] and in Kuwait [22]. CoNS have been also reported as the main causative factor for early-onset neonatal infections in Canada [24], USA [27], and China [28].

A number of mobile phones in our study were found to be colonized with potentially pathogenic bacteria, namely MRSA and Gram-negative bacteria. MRSA was identified in three (1.4 %) mobile phones, none were resistant to vancomycin, which is the drug of choice for MRSA infections [29]. The rate of MRSA contamination of clinicians’ mobile phones is slightly lower in our study compared to other literature, in which it ranged from 1.9 % to 10.3 % [8, 10]. *Acinetobacter* species have been frequently identified as a cause of widespread hospital outbreaks, including those in ICUs [30]. Gram-negative bacteria were identified in 15 (7.0 %) mobile phones, of which six (2.8 %) were *Acinetobacter* species; but none were resistant to meropenems. The rate of contamination with *Acinetobacter* species is consistent with other studies, which reported that between 1 % and 12 % of clinicians’ mobile phones were contaminated by *Acinetobacter* species [8]. Another organism identified in our study was *Pseudomonas stutzeri*, which was found to be sensitive to gentamicin and amikacin. In addition, *Escherichia coli* was isolated from one mobile phone, which suggests low level of mobile phone hygiene and hand hygiene since this organism is part of the intestinal flora; and among the leading causes of HAIs.

In terms of self-reported mobile phone hygiene practices, 66.5 % of the participants have never disinfected their mobile phones. This is similar to that reported from Saudi Arabia, where 76.0 % of clinicians have never disinfected their mobile phones [18]; and in a surgical setting in Northern Ireland, where only 37 % of healthcare workers admitted cleaning their mobile phone regularly [31]. Our study showed that clinicians who have ever disinfected their mobile phones were less likely to have contaminated mobile phones compared to clinicians who have never disinfected their mobile phones, and this was statistically significant after adjusting for potential confounders. No other factors was significantly associated with mobile phone contamination in our study. Other studies have investigated factors related to mobile phone contamination and included gender of the clinician, number of times the mobile phone is used at work, type of phone, and medical specialty of the clinician; but none of these factors was found to be significant [5, 8, 10, 12].

Previous studies have demonstrated that the microbiological profile of the clinicians’ mobile phones correlates with the pathogens isolated from the clinicians’ hands, which may indicate that mobile phone contamination might be a predictor for hand contamination [21, 32], and hence hand hygiene. However, we did not find an association between mobile phone contamination and clinicians’ self-reported hand hygiene practices in our study. Previously, it has been demonstrated that observed hand hygiene practices are unrelated or weakly correlated to self-reported hand hygiene [33–35], which may explain our findings.

In our study, we investigated the opinion of clinicians about the potential role of mobile phones in spreading nosocomial infections. Approximately, 63.0 % of clinicians thought that mobile phones can play a role in spreading infections in healthcare settings. However, 68.0 % of clinicians opposed banning the use of mobile phones in their units. This is slightly lower than what has been reported in a study from UK, in which 78.0 % of clinicians opposed banning the use of mobile phones in hospitals [9]. While losing the momentum to ban mobile phones in ICUs and other clinical settings, it is sensible to increase the awareness about mobile phones disinfection rather than trying to forcefully ban using mobile phones in clinical settings.

This is the first study that investigated the prevalence of contamination of clinicians’ mobile phones and their microbiological profile in all ICUs, PICUs, and NCUs in public secondary care hospitals in Kuwait. We have attempted to enroll all clinicians in ICUs, PICUs, and NCUs, and only 3.8 % of those approached refused to participate. However, of 229 eligible clinicians who were not officially on leave, 18 were not approachable despite the four attempts we made to recruit them (Fig. 1). Nevertheless, there is no obvious reason to assume that mobile phone contamination and mobile phone disinfection practices would be different between those who participated and those who did not. It is possible that some clinicians had disinfected their mobile phones when they overheard about the study and before they were approached to participate (Hawthorne effect). This, if exists, will underestimate the microbiological contamination rate and will also make a fallacious association between self-reported mobile phone disinfection and mobile phone contamination.

## Conclusion

The prevalence of clinicians’ mobile phones that are contaminated by various microorganism in the ICUs, PICUs, and NCUs was high. Although most microorganisms can be considered non-pathogenic in normal circumstances, these are potentially harmful in ICU and NCU settings, where patients are extremely vulnerable to infections. Some mobile phones harbored extremely harmful bacteria, such as MRSA or Gram-negative organisms. Kuwait, like other countries in the Gulf region, has introduced a sophisticated tertiary care but probably without considerable effort to reduce infections associated with these services. Our findings highlight the need for a more comprehensive approach to reduce nosocomial infections, which in addition to promoting hand hygiene also focus on cleanliness of mobile phones and other objects that clinicians may carry. Only minority of clinicians have ever disinfected their mobile phones, which is not an optimal practice and highlights the need to increase the awareness about mobile phones disinfection among clinicians, given that banning mobile phones in ICU settings is losing momentum. Finally, further research is needed in order to provide evidence that better mobile phone hygiene will lead to a reduction in HAIs.
